# Soft tissue Rosai–Dorfman disease in child: A case report and literature review

**DOI:** 10.1097/MD.0000000000004021

**Published:** 2016-07-22

**Authors:** Yunlan Xu, Bingqiang Han, Jie Yang, Jing Ma, Ji Chen, Zhigang Wang

**Affiliations:** a Department of Pediatric Orthopedics; b Department of Pathology, Shanghai Children's Medical Center, Shanghai Jiaotong University School of Medicine, Shanghai; c Department of Pediatric Orthopedics, Chengdu Women's and Children's Center Hospital, Chengdu, Sichuan Province, China.

**Keywords:** child, MRI, Rosai–Dorfman, soft tissue

## Abstract

**Background:**

Rosai–Dorfman disease was commonly characterized as massive, painless, bilateral, symmetric cervical lymphadenopathy, with fever, leukocytosis, and elevated sedimentation rate. However, soft tissue Rosai–Dorfman disease (STRDD) is a rare benign tumor.

**Methods:**

We hereby present 1 case of a 17-month-old girl, an isolated subcutaneous mass was detected on her right forearm, and no signs of pain, swelling, or erythema were observed at the site.

**Results:**

The patient underwent an excisional biopsy for the mass. Immunohistochemistry results showed that it was positive for S-100 protein and CD68, whereas negative for CD1a, which supported the diagnosis of STRDD. Conclusions: The patient showed no evidence of recurrence or metastasis 2 years after the surgery.

Some multifocal masses were reported to be much more prone to recurrence. Further follow-up of STRDD is necessary.

## 1 Introduction

Sinus histiocytosis with massive lymphadenopathy (SHML), also known proverbially as Rosai-Dorfman disease (RDD), was first described by Rosai and Dorfman in 1969,^[1]^ which was commonly characterized as massive, painless, bilateral, symmetric cervical lymphadenopathy, with fever, leukocytosis, elevated sedimentation rate, and hyper-γ-globulinemia. Immunophenotypic studies have supported the interpretation that RDD cells were part of the mononuclear phagocyte and immunoregulatory effector system, belonging to the macrophage/histiocytic family.^[2]^ Found worldwide and affecting individuals predominantly with mean onset age of 20.6 years,^[2]^ RDD is slightly more common in men (1.4:1) and is significantly more common among whites and blacks than Asians.^[3]^

Extranodal sites are often involved with skin, central nervous system, upper respiratory system, long bones, and soft tissue (43% of cases in registry database).^[2]^ Deeply soft tissue Rosai-Dorfman disease (STRDD) is rare, with sporadic cases previously reported in no >3% patients. The ethics committee of the Shanghai children's Medical Center reviewed and approved this study. Written, informed consent was obtained from the patients. We hereby report one case of RDD on the forearm and review the literature.

## 2 Case report

### 2.1 Clinical features

A 17-month-old girl was admitted into our hospital for an isolated subcutaneous nodule (mass) on the right forearm, no signs of pain, swelling, or erythema were observed at the site, and it was enlarging gradually in the recent 3 months. The girl was born in Shanghai and was usually healthy. Physical examination on admission showed an isolated superficial 2.0 × 1.5 cm soft tissue mass on distal right forearm, it was soft, movable; and nontender, full-range movement was observed on the elbow and wrist.

**Figure 1:**
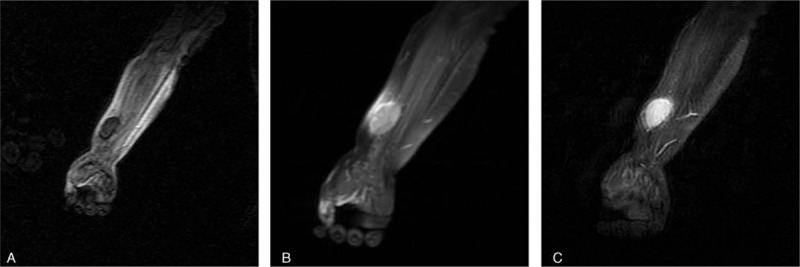
MRI of STRDD. (A) Note that lesion has a low signal intensity on T1-weighted image. (B) High signal intensity on T2-weighted MRI. (C) After injection of gadolinium contrast agent, a strong enhancement can be noted.

Results of laboratory tests were as follows: peripheral while blood cell count 7.9 × 10^9^ cells/L, serum C-reactive protein (CRP) <1 mg/L, and erythrocyte sedimentation rate (ESR) 12 mm/hour, without any abnormal findings. Human herpesvirus-6 (HHV-6)-specific DNA sequences by PCR test was also negative. Magnetic resonance imaging (MRI) scan (slice 30. thickness 5.0 mm, gap 1.0 mm) of the mass showed medial signal intensity on T1-weighted (TR/TE 450/35 ms) and high signal intensity on T2-weighted images (TR/TE 2500/100 ms), with strong enhancement after injection of gadolinium contrast agent. However, fat-suppressed showed high signal intensity on T1-weighted and high signal intensity on T2-weighted images, with strong enhancement after the injection of gadolinium contrast agent (Fig. 1 A–C.

### 2.2 Pathological features

**Figure 2:**
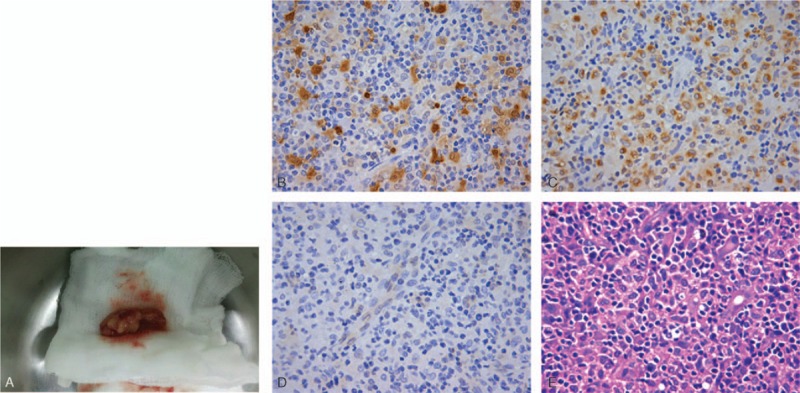
Note that a Grossly STRDD lesion was tan-pale and soft, circumscribed and subcutaneoust (A). The histocytes of STRDD are immunohistochemically positive for S-100 protein and CD68, and negative for CD1a (B, C, D ×200). Additionally, lymphocyte phagocytosis (emperipolesis) is noted (arrow, E ×200).

The patient underwent an excisional biopsy followed by pathologic examination on day 3 after hospitalization. The lesion was soft tissue in size of 1.5 × 0.8 × 0.5 cm, with irregular shape in tan-pale color (Fig. 2AA). Histologically, sections of HE stain showed a diffused infiltration of large histiocytes, lymphocytes, and plasma cells with scattered neutrophils. The histiocytes showed abundant pale eosinophilic cytoplasm and mildly atypical round vesicular nuclei. Immunohistochemical results were positive for S-100 protein, CD68, and negative for CD1a (Fig. 2B, C, D). Moreover, immunohistochemical stains for monoclonal cytokeratin 7, cytokeratin 20, epithelial membrane antigen (EMA), myeloperoxidase (MPO), calretinin, mesothelial cell, actin, desmin, human melanoma black-45 (HMB-45), melanoma, CD3, CD15, CD30, CD31, and anaplastic lymphoma kinase (ALK) were all negative. Notably, lymphocyte phagocytosis (emperipolesis) was detected (Fig. 2E).

### 2.3 Follow-up and outcomes

The patient was diagnosed as having STRDD of the forearm and was discharged from hospital on day 4 after the surgery. The girl has been followed up for 2 years and no recurrence or metastasis has been observed.

## 3 Discussion

Until now, no >1000 RDD cases have been reported in English journals.^[4]^ It is often accumulated in extranodal sites including the orbit, eyelid, skin, bone, central nervous system, and soft tissues. However, simple soft tissue manifestation of RDD (without lymphadenopathy or other systemic symptoms) is rarely seen, which occurs in <3% of patients.^[5–9]^

STRDD is primarily found in trunk and proximal extremities as a rapidly evolutional entity. On occasion, it manifests as a multifocal and persistent disease. Although RDD is slightly more common in men,^[3]^ STRDD has a female sex predominance of nearly 3:1, also with a broader size range and a wider age range, which was supported by previous literatures.^[10–12]^ The study of Al-Daraji et al^[12]^ showed that multifocal STRDD was much more prone to recurrence. Owing to its low incidence, there was no difference in recurrence between males and females. Our patient, with an isolated mass on the forearm, has been followed up for 2 years postoperatively, without any sign of recurrence. To our knowledge, this is the first case report of STRDD located on the forearm in children.

Laboratory tests and radiograph results were unremarkable. Noguchi et al^[13]^ reported that patients of RDD might show slight elevation of CRP and ESR. However, such results were not observed in our case. Laboratory parameters may show nonspecific increase in RDD, which was reported by a previous literature.^[4]^

The diagnosis of STRDD is mainly confirmed by pathological examinations. Specimens are mainly obtained by open surgical biopsy or fine needle aspiration. In general, histopathological inspection markedly shows a large number of mixed cell population, including mature plasma cells and lymphocytes.^[1]^ The most typical cells are histiocytes of accentuated phagocytic appearance. The most useful markers of histiocytes in RDD are positive for S-100 protein and CD68, and negative for CD1a.^[14]^ In our case, immunohistochemical stains for monoclonal cytokeratin 7, EMA, MOP, calretinin, mesothelial cell, actin, desmin, HMB-45, melanoma, CD3, CD15, CD30, CD31, and ALK were all negative. Lippi et al^[15]^ showed the presence of HHV-6-specific DNA within histiocytes of some RDD patients, which therefore indicated that Epstein–Barr virus (EBV) might play a role in the onset of RDD. However, HHV-6 is so commonly present in lymphoid tissue that significance of this finding remains dubious. HHV-6-specific DNA test by PCR was also negative in our case.

Differential diagnosis of RDD includes histiocytosis of Langerhans cells, histiocytic sarcoma, lysosomal storage diseases (eg, Gaucher disease), classical Hodgkin lymphoma, melanoma and metastatic carcinomas, and infections caused by Histoplasma and mycobacteria involving lymph nodes. Immunohistochemical staining for S-100 and CD68 is helpful in distinguishing RDD from diseases mentioned above.^[4]^

Owing to its low incidence, no ideal or standard treatment has been defined for STRDD. The predilection sites of the lesion and its self-limiting nature also make the majority of RDD patients not necessary to be intervened. Nevertheless, the course of RDD is still unpredictable. When vital organs are involved, interventions proposed by previous literatures include corticosteroids administration, chemotherapy, radiotherapy, and surgical resection, but their efficacy remains uncertain.^[16]^ In our case, the girl has been followed up for 2 years after the surgery and no recurrence has been observed.

## 4 Conclusion

In conclusion, we presented a rare case of STRDD in children. Simple STRDD is an unknown benign neoplasm and is mainly confirmed by pathological examinations, showing positive for S-100 protein and CD68, and negative for CD1a. Furthermore, follow-up of STRDD is necessary.
